# Editorial to the Special Issue “Total Synthesis of Natural Products: A Themed Issue Dedicated to Professor Dr. Dieter Schinzer for His 65th Birthday”

**DOI:** 10.3390/molecules25245841

**Published:** 2020-12-10

**Authors:** Ari M. P. Koskinen

**Affiliations:** Department of Chemistry and Materials Science, Aalto University School of Chemical Engineering, Kemistintie 1, P.O. Box 16100, 02150 Espoo, Finland; ari.koskinen@aalto.fi

Natural products have intrigued humans throughout history. Plants with physiological activities, fermentation products, extracts with aroma, scent, or other properties have catalyzed the development of physical methods of separation of compounds and eventually the chemical synthesis of compounds. It is no coincidence that the first synthesis of an organic compound was that of a natural product. Since Wöhler’s synthesis of urea (no stereocenters) nearly two centuries ago, the synthesis of natural products has evolved through Komppa’s synthesis of camphor (one independent stereocenter) a century ago to a stage where compounds of enormous complexity can be attained through chemical synthesis (e.g., palytoxin with 64 stereocenters by Kishi in 1994). This development continues undauntedly, and it stimulates the invention of new synthetic reactions, new technological inventions, and new strategic thinking. The synthesis of natural products also stimulates the minds of medicinal chemists to develop ever better pharmaceutical products inspired by nature.

Professor Dieter Schinzer had his initial training in organic synthesis with eminent mentors (Professors Manfred Reetz, Clayton Heathcock and Ekkehard Winterfeldt). Despite his wide research interests in organometallic chemistry (especially silicon, tin and manganese), synthetic methodology and medicinal chemistry, Dr. Schinzer is first and foremost a devoted natural product chemist. In 1997, his research group was among the first to achieve the total synthesis of epothilones. His interest in medicinal chemistry led him to be a founder and Chief Scientific Officer (CSO) of Molecular Links Sachsen-Anhalt GmbH (MOLISA, Berlin, Germany) in 2002. He has been instrumental in developing several European COST research consortia under the general umbrella of Bioactive Natural Product synthesis. He also served as the Chairman of the COST Domain Committee for Chemistry and Molecular Sciences from 2006 to 2009. Within these activities, Professor Schinzer initiated a highly successful series of ”ESF-COST High-Level Research Conference Natural Products Chemistry, Biology and Medicine” held at Acquafredda di Maratea, Italy. This issue highly reflects the atmosphere in those meetings.

Eleven of the twelve papers in this issue deal with syntheses of natural products, six being original research articles and five reviews on various aspects of synthesis. The final paper in the issue provides an interesting but very important, although nowadays relatively rare, viewpoint on biogenetic considerations.

Rhizoxins are 16-membered macrolides responsible for the rice seedling blight, a plant disease that leads to cell cycle arrest and ultimately death of the plant. Rhizoxins are produced by an endosymbiotic bacteria of the genus *Burkholderia* and passed on to the plant by a *Rhizopus* fungus. The research group of Karl-Heinz Altman has a longstanding interest in macrocyclic natural products, and in [1] presented they detail carefully various synthetic approaches based on ring closing metathesis (RCM) targeting rhizoxin F. However, the RCM approaches eventually failed. Ring closing alkyne metathesis (RCAM) pioneered by Fürstner eventually came to rescue and rhizoxin F was obtained in ≈1.5% overall yield from 1,4-butanediol in a sequence of 24 steps in the longest linear sequence. Additionally, a highly *Z*-selective reduction method for alkynes was developed.

Food and feedstock can be contaminated with fungi that secrete toxic secondary metabolites known as mycotoxins. Those produced by *Alternaria* fungi are frequently found in agricultural crops as well as soil, wallpapers, and textures, and have been implicated in several animal and human health ailments. The paper by Sebald et al. [2] describes the synthesis of (−)-altenuene in trideuterated form to be used as an internal standard in stable isotope dilution analysis (SIDA) LC/MS-MS method for mycotoxins. The synthesis is based on an earlier reported synthesis of the natural product, and through successful optimization of the synthesis route the deuterium labeled natural product was obtained with an overall yield of 17% after nine steps from inexpensive commercially available d-(−)-quinic acid. Its applicability as an internal standard for SIDA was also demonstrated.

Paraphaeosphaerides are recently isolated natural products which exhibit inhibitory activity against experimental breast cancer cells including cisplatin resistant cells. They are, therefore, considered potential lead compounds for anticancer drug discovery. The communication by Kobayashi et al. [3] describes the synthesis of the proposed structure of paraphaeosphaeride C following a synthesis route similar to the earlier synthesis of a related congener by the same group. The synthesis described in this paper featured a Knochel-Hauser base (TMPMgCl·LiCl)-mediated intramolecular vinyl anion aldol reaction and subsequent appropriate functional group transformations, including regioselective methylenation and stereoselective reduction to establish a critical stereocenter. Careful extensive NMR studies showed that the data for the synthetic compound did not match those of the natural product. Additionally, the optical rotation of the synthetic material was opposite in sign and substantially different in magnitude to that of the natural product. This work thus implies that the structure of natural paraphaeosphaeride C has been incorrectly assigned.

Of the three types of opioid receptors (µ, δ, κ), the κ-opioid receptor is considered especially interesting as its activation produces analgesia with minimal physical dependence. The Hudlicky research group has a long history of investigating chemoenzymatic synthesis of morphinans. In this paper [4], they describe an efficient synthesis of (+)-10-keto-oxycodone with a total of 14 operations and an overall yield of about 2%. The absolute stereochemistry was secured via a whole cell fermentation with *Escherichia coli* JM109 (pDTG601A) enzymatic dihydroxylation of phenethyl acetate to the corresponding *cis*-cyclohexadienediol. Subsequent reactions were involved an intramolecular Heck cyclization, samarium iodide mediated pinacol type coupling, and a CAN-mediated functionalization to give a key azidonitrate.

Uncontrolled excessive acute inflammation, which by nature is self-resolving, and chronic unresolved inflammation may develop into several diseases, including cardiovascular diseases, cancer, rheumatoid arthritis, and neurological disorders—e.g., Parkinson’s disease and Alzheimer’s disease. It is, therefore, of utmost importance to understand the processes inducing the resolution phase of inflammation and homeostasis (return to the normal physiology). A number of endogenously formed compounds have been coined specialized pro resolving mediators (SPMs), and in this paper [5], Hansen’s research group describes the synthesis, structural confirmation, and biosynthesis of 22-OH-PD1_n-3 DPA_. The key reaction in the synthesis is a Sonogashira coupling of a vinyl bromide with a terminal alkyne followed by *Z*-selective Boland reduction of the triple bond. Subsequent cellular studies revealed that 22-OH-PD1_n-3 DPA_ is formed from n-3 docosapentaenoic acid in human serum and that 22-OH-PD1_n-3 DPA_ is a secondary metabolite produced by ω-oxidation of PD1_n-3 DPA_ in human neutrophils and in human monocytes.

Cornexistin and hydroxycornexistin are structurally unique fungal natural products containing a nine-membered carbocycle fused to a maleic anhydride. The compounds were isolated in the late 1980s and early 1990s from a culture of *Paecilomyces variotii* Bainier, and their potent and selective herbicidal activities attracted the attention of the agrochemical industry. The maleic anhydride substructure makes the compounds prone to rapid degradation on exposure to light, acid, or base. Stephen Clark’s research group has developed a short convergent approach to the synthesis of the core framework of these fascinating compounds employing an intramolecular Nozaki–Hiyama–Kishi reaction as the key reaction [6]. Despite the difficulty in the formation of the 9-membered ring, the cyclization proceeded with good yield (43%). The ring closure precursor was obtained through a palladium mean mediated sp^2^–sp^3^ coupling of a vinyl stannane with a chlorinated furan derivative.

Pyranones with a long carbon chain at carbon atom 6 are natural compounds, which exhibit varied biological activities including anticancer, antiviral, antifungal, antituberculotic, and antimicrobial effects. The review by Avula et al. [7] sums up the stereoselective syntheses of ten of these intriguing natural products. Many of the compounds have surrendered to several syntheses within the past decade or so; for instance, there are nine syntheses of dodoneine and 12 syntheses of rugulactone. For introduction of the chirality in the molecules, enantioselective allylation as well as the use of chiral pool starting materials are the most prominent approaches. Ring closing metathesis finds a special role for the formation of the pyranone ring and cross metathesis is prominent for the introduction of the sidechains. The value of total synthesis in proving the structures of isolated natural product is again highlighted by the fact that the structures of synargentolide A and passifloricin A have been corrected on the basis of syntheses.

The Pictet–Spengler reaction (PS) is an efficient versatile synthetic method for the construction of privileged pharmacophores including tetrahydroisoquinolines and tetrahydro-β-carbolines. The review by Calcaterra et al. [8] highlights the developments within a five-year period (2011–2015) in great detail (313 references, 89 Schemes). The review covers applications in total synthesis of complex alkaloids, in combination with chiral catalysts as well as for the generation of libraries of compounds in medicinal chemistry. The PS reactions in combination with ring closing metathesis, isomerization, Michael addition, and gold- or Brønsted acid-catalyzed N-acyliminium cyclizations are covered, as well as Ugi multicomponent reactions. Syntheses of peptidomimetics, synthetic heterocycles, and natural compounds are covered, and finally, the enzymatic versions of PS are described for biosynthesis, biotransformations, and bioconjugations.

Enantioselective synthesis of complex bioactive natural products and pharmaceuticals has gained importance especially within the last four decades. During the past two decades, organocatalytic reactions have become prominent, and the review by Wang [9] details those achieved using chiral secondary amine catalyzed reactions of α,β-unsaturated aldehydes. Friedel Crafts reactions, Michael additions (in their oxa, aza, and normal versions), hydrogenation reactions, and cycloadditions (cyclopropanations, epoxidations, Diels-Alder reactions including IMDA, [3 + 2]- and [3 + 3]-cycloadditions) are covered with extensive examples. Finally, asymmetric organocatalytic cascade reactions are highlighted in the synthesis of a lignan compound and several alkaloids. Impressive examples are highlighted, such as the shortest asymmetric total synthesis of frondosin B, industrial scale asymmetric total synthesis of MK-0974, organocascade and collective asymmetric total syntheses of strychnine, and other indole alkaloids. Significant challenges remain, however, for instance in the industrial scale application in enantioselective drug synthesis.

Arbutin is the β-d-glucopyranoside of hydroquinone, which occurs in the leaves of a number of medicinal plants—for example, in “bearberries” of the *Ericaceae* and *Saxifragaceae* families. The leaves of these plants have been traditionally used as folk medicines both in China and the Americas, especially for wound healing and treatment of urinary tract infections. The review by Zhao et al. [10] covers the previous syntheses of arbutin as well as its biosynthesis. The review culminates in biocatalytic synthesis of arbutin through a one pot production which circumvents the use of hydroquinone. This unusual approach starts with benzene which is first chemo- and regioselectively dihydroxylated with a mutant cytochrome P450-BM3. The in situ formed hydroquinone is glycosylated by an appropriate arbutin synthetase. The one pot whole cell cascade in *E. coli* leads from benzene to arbutin in yields of 80–83%.

The vinylogous Mukaiyama aldol reaction (VMAR) is a powerful transformation as it allows the synthesis of larger carbon frameworks containing a double bond between the carbonyl and hydroxy functionalities. The reaction efficiently generates two new stereocenters. The review by Cordes and Kalesse [11] gives a comprehensive introduction to this important transformation. A pedagogically sound introduction gives the reader adequate understanding to follow the ensuing examples. Both enantioselective and diastereoselective variants of the reaction are discussed using a number of examples from natural product syntheses.

Mucosin is a C_20_-compound related to arachidonic acid. It was isolated in 1997 from the Mediterranean sea sponge *Reniera mucosa*, and the structure of its methyl ester was elucidated by spectroscopic means. Stereocontrolled total synthesis by the authors of the present paper showed that the proposed structure was incorrect. In this paper, Nolsøe et al. [12] consider different biosynthetic pathways from arachidonic acid to mucosin, including both concerted and stepwise processes. The authors conclude that mucosin does not share its biogenetic origin with polyketide marine natural products containing the *trans*-bicyclo[4.3.0]non-2-ene core but is derived from polyunsaturated fatty acids through a different route.

## References

[1] Liniger M., Neuhaus C.M., Altmann K.-H. (2020). Ring-Closing Metathesis Approaches towards the Total Synthesis of Rhizoxins. Molecules.

[2] Sebald M.A., Gebauer J., Sommerfeld T., Koch M. (2019). First Synthesis of (−)-Altenuene-D3 Suitable as Internal Standard for Isotope Dilution Mass Spectrometry. Molecules.

[3] Kobayashi K., Kunimura R., Kogen H. (2019). Total Synthesis of the Proposed Structure of Paraphaeosphaeride C. Molecules.

[4] Endoma-Arias M.A., Dela Paz H., Hudlicky T. (2019). Chemoenzymatic Total Synthesis of (+)-10-Keto-Oxycodone from Phenethyl Acetate. Molecules.

[5] Nesman J.I., Gangestad Primdahl K., Tungen J.E., Palmas F., Dalli J., Hansen T.V. (2019). Synthesis, Structural Confirmation, and Biosynthesis of 22-OH-PD1n-3 DPA. Molecules.

[6] Aimon A., Farrugia L.J., Clark J.S. (2019). Synthesis of the Core Framework of the Cornexistins by Intramolecular Nozaki-Hiyama-Kishi Coupling. Molecules.

[7] Avula S.K., Das B., Csuk R., Al-Rawahi A., Al-Harrasi A. (2020). Recent Advances in the Stereoselective Total Synthesis of Natural Pyranones Having Long Side Chains. Molecules.

[8] Calcaterra A., Mangiardi L., Delle Monache G., Quaglio D., Balducci S., Berardozzi S., Iazzetti A., Franzini R., Botta B., Ghirga F. (2020). The Pictet-Spengler Reaction Updates Its Habits. Molecules.

[9] Wang Z. (2019). Advances in the Asymmetric Total Synthesis of Natural Products Using Chiral Secondary Amine Catalyzed Reactions of α,β-Unsaturated Aldehydes. Molecules.

[10] Zhou H., Zhao J., Li A., Reetz M.T. (2019). Chemical and Biocatalytic Routes to Arbutin. Molecules.

[11] Cordes M., Kalesse M. (2019). Very Recent Advances in Vinylogous Mukaiyama Aldol Reactions and Their Applications to Synthesis. Molecules.

[12] Nolsøe J.M.J., Aursnes M., Stenstrøm Y.H., Hansen T.V. (2019). Some Biogenetic Considerations Regarding the Marine Natural Product (−)-Mucosin. Molecules.

